# Mutually exclusive lymphangiogenesis or perineural infiltration in human skin squamous-cell carcinoma

**DOI:** 10.18632/oncotarget.27915

**Published:** 2021-03-30

**Authors:** Julien Schaller, Hélène Maby-El Hajjami, Sylvie Rusakiewicz, Kalliopi Ioannidou, Nathalie Piazzon, Alexandra Miles, Déla Golshayan, Olivier Gaide, Daniel Hohl, Daniel E. Speiser, Karin Schaeuble

**Affiliations:** ^1^Department of Oncology, University Hospital Lausanne (CHUV/UNIL), Lausanne, Switzerland; ^2^Institute of Pathology, University Hospital Lausanne (CHUV), Lausanne, Switzerland; ^3^Department of Dermatology, University Hospital Lausanne (CHUV), Lausanne, Switzerland; ^4^Transplantation Center, University Hospital Lausanne (CHUV/UNIL), Lausanne, Switzerland

**Keywords:** lymphatic vessel, CD8+ T cell, perineural infiltration, skin squamous-cell carcinoma, tumor immunology

## Abstract

Although tumor-associated lymphangiogenesis correlates with metastasis and poor prognosis in several cancers, it also supports T cell infiltration into the tumor and predicts favorable outcome to immunotherapy. The role of lymphatic vessels in skin squamous-cell carcinoma (sSCC), the second most common form of skin cancer, remains mostly unknown. Although anti-PD-1 therapy is beneficial for some patients with advanced sSCC, a greater understanding of disease mechanisms is still needed to develop better therapies.

Using quantitative multiplex immunohistochemistry, we analyzed sSCC sections from 36 patients. CD8+ T cell infiltration showed great differences between patients, whereby these cells were mainly excluded from the tumor mass. Similar to our data in melanoma, sSCC with high density of lymphatic endothelial cells showed increased CD8+ T cell density in tumor areas. An entirely new observation is that sSCC with perineural infiltration but without metastasis was characterized by low lymphatic endothelial cell density. Since both, metastasis and perineural infiltration are known to affect tumor progression and patients’ prognosis, it is important to identify the molecular drivers, opening future options for therapeutic targeting. Our data suggest that the mechanisms underlying perineural infiltration may be linked with the biology of lymphatic vessels and thus stroma.

## INTRODUCTION

Over the last few years, oncology research is increasingly focused on the understanding of mutual interactions between cancer, stromal and immune cells in the tumor microenvironment (TME). Immune cells have been shown to play various roles impacting tumor progression, metastasis formation and responsiveness to chemo- and immunotherapies [1–3]. However, the so-called immune-checkpoint blockade therapy is successful in only some patients, thereby raising the question: what are the major determinants of an effective immune response?

A major characteristic of many tumors in both humans and mice is the proliferation and activation of lymphatic endothelial cells (LECs) within the primary tumor, as well as in pre-metastatic and metastatic sites [4–6]. Specific growth factors like vascular endothelial growth factor (VEGF) –C and –D secreted by tumor cells and/or immune cells stimulate lymphangiogenesis in the vicinity of the tumor [4]. The resulting increased lymphatic vessel density (LVD) correlates with tumor metastasis formation in murine models of melanoma, breast and pancreatic cancers [7]. Lymphatic vessels offer a route for cancer cells to disseminate, enhanced by secretion of different chemokines such as CCL21 that actively supports tumor cell invasion into lymphatic vessels and promotes lymph node (LN) metastases [8].

The second most common cutaneous cancer, skin squamous-cell carcinoma (sSCC), remains mostly localized and non-aggressive. However, it can occasionally metastasize into LNs and to distant organs, as well as spread along nerves. Metastases have been shown to occur in 4–5% of sSCC cases, with the major prognostic factors being the tumor thickness, the increased horizontal tumor size and the immune suppression status of the patient [9–12]. In addition, the presence of perineural infiltration in the tumor is a bad prognostic factor, which is often associated with increased loco-regional recurrence and decreased patient survival [11, 13]. Investigations into the immune cell composition showed the presence of various immune cells in sSCC. Interestingly, in murine sSCC the beneficial role of CD8+ T cells in tumor control varied between the type of model [14]. Similar to other cancers, CD8+ T cells found in metastatic sSCC express high levels of immune inhibitory receptors like Tim-3 and programmed death-1 (PD-1) hindering tumor clearance. Furthermore, programmed cell death-ligand 1 (PD-L1) expression correlates with metastases in sSCC patients [15].

Lymphatic vessels have a dual role, either supporting or hindering immune responses [16]. Indeed, LECs can produce immunosuppressive molecules such as nitric oxide (NO) and indoleamine-2,3-dioxygenase (IDO) that interfere with the activation and proliferation of T cells [17, 18]. In addition, antigen cross-presentation and PD-L1 expression by LECs may promote immune tolerance [19, 20]. On the other hand, the presence of lymphatic vessels in the TME has also been shown to support adaptive anti-tumoral immune responses by carrying immune cells and antigens to the draining LNs [21]. Further, they can promote T cell recruitment to the TME via CCL21/CCR7 signaling [22]. We have previously shown that LVD in the TME of human melanoma positively correlates with tumor infiltrating CD8+ T cells [5] and recent findings in a melanoma mouse model and a clinical immunotherapy trial demonstrated that the presence of LECs increased the likelihood of immunotherapy success through their support of T cell responses [22].

Increased LVD has been linked to poor clinical outcome in patients with various cancers, including melanoma [7, 23]. In sSCC, upregulation of VEGF-C expression by macrophages and higher LVD correlate with metastasis formation [24, 25]. Together, these data suggest that the frequency of LECs in the TME may promote sSCC aggressiveness and may play a central role in shaping immune cell infiltration in this type of skin cancer. For further investigation of possible associations of lymphatic vessels with stroma and T cells, we performed a comprehensive analysis of the abundance and localization of lymphatic vessels in 36 patients with sSCC.

## RESULTS

### Multiplex immunohistochemistry analysis reveals a distinct distribution of CD8+ T cells and lymphatic endothelial cells within the TME of human sSCC tissues

**Figure 1:**
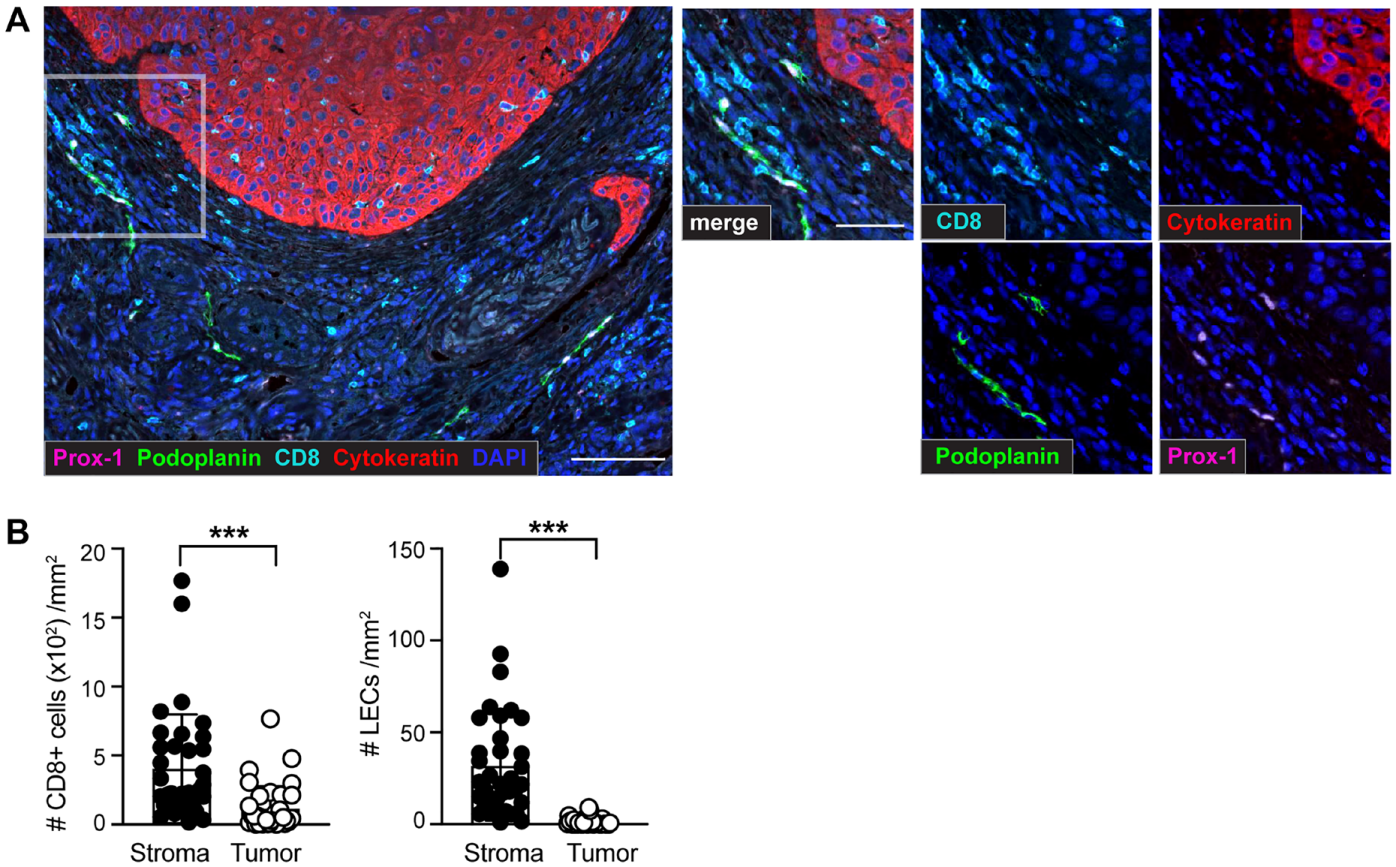
Identification of CD8+ T cells and lymphatic endothelial cells (LECs) within the tumor microenvironment (TME) of sSCC sections using multiplex immunohistochemistry (IHC). Formalin-fixed paraffin-embedded (FFPE) tissue sections of 36 primary sSCC samples were investigated using multiplex IHC followed by quantitative image analysis. (**A**) Representative images highlighting CD8+ T cells (CD8+), LECs (identified by co-labeling of Prox-1+ and Podoplanin+) and tumor cells (Cytokeratin+) in primary sSCC. Scale bar = 100 μm; zoom-in scale bar = 50 μm. (**B**) Number of CD8+ T cells and LECs per mm^2^ stroma and tumor areas. *n* = 36. Bar graphs showing mean ± SD. ^***^ *p* < 0.001.

The appearance of metastases in patients with sSCC has recently been associated with increased intratumoral lymphatic vessel density (LVD) [24] but it remains unknown whether the abundance of lymphatic vessels correlates with immune cell infiltration in the tumor. In order to investigate lymphatic vessels and CD8+ T cells in patients with sSCC we developed a multiplex immunohistochemistry protocol to study lymphatic endothelial cells (LECs) in large fields of human sSCC tissue sections (Figure 1A). Using a trainable image analysis software, tumor areas were defined by cytokeratin-positive labeling whereas all cytokeratin-negative zones were determined as stroma. Tumor cells, LECs and CD8+ T cells were identified based on the expression of cell type specific markers followed by analysis using the phenotyping module (Supplementary Figure 1A). As there is no single marker that identifies lymphatic vessels specifically in human tumor tissues, we identified LECs by co-staining of Prox-1 and Podoplanin. To validate the image analysis method, we compared the trainable phenotyping of LECs with a visual identification of lymphatic vessels. We observed a strong positive correlation between the manual quantification and the number of Prox-1+ Podoplanin+ LECs quantified by phenotyping (Supplementary Figure 1B), validating our method to specifically identify LECs and confirming that quantification of LECs correlated with the abundance of lymphatic vessels in a tissue section.

To define and locate lymphatic vessels and CD8+ T cells, we investigated large-scale tissue sections from a cohort of 36 patients (Table 1). A quantitative analysis of the cellular distribution showed heterogeneous localization of LECs and CD8+ T cells across all sSCC sections. Similar to previous findings in other tumor types [26], our results showed a significantly higher density of both, LECs and CD8+ T cells, in the stroma compared to the sSCC tumor mass (Figure 1B). Focusing on the distribution of CD8+ T cells within the primary tumor tissue we found a significant positive correlation between the density of stroma and tumor infiltrating CD8+ T cells (Supplementary Figure 1C). Interestingly, and in contrast to CD8+ T cells, LECs were found almost exclusively in the stroma surrounding the tumor and were absent in the tumor mass. Hereafter, the LEC density will always be referred to as the LEC density in the stroma (labeled as per mm^2^ stroma).

**Table 1:** Patient characteristics

		entire cohort	w/o PNI	with PNI	*P*-value
	*n*	36	20	16	
Gender, *n* (%)	Woman	11 (30.6)	8 (40)	3 (19)	
	Men	25 (69.4)	12 (60)	13 (81)	
Age at diagnosis	years (mean ± SD)	72.5 ± 15.3	70.3 ± 11.2	75.2 ± 19.3	
	Range of age	40–102	40–87	40–102	
Iatrogenic immunosuppression, *n* (%)	Yes	11 (30.5)	6 (30)	5 (31.2)	
	No	24 (66.7)	13 (65)	11 (68.8)	
	NA	1 (2.8)	1 (5)	0 (0)	
Localization, *n* (%)	upper/lower extremities	6 (16.7)	3 (15)	3 (18.8)	
	trunk	3 (8.3)	2 (10)	1 (6.2)	
	head	27 (75)	15 (75)	12 (75)	
Metastasis (%)	Yes	38.89			
	No	61.11			
Differentiation, *n* (%)	Well	7 (19.4)	5 (25)	2 (12.5)	^*^ *p = 0.04*
	Moderate to Well	3 (8.3)	0 (0)	3 (19)	
	Moderate	10 (27.8)	8 (40)	2 (12.5)	
	Poor to Moderate	5 (13.9)	2 (10)	3 (19)	
	Poor	8 (22.2)	2 (10)	6 (37)	
	NA	3 (8.3)	3 (15)	0 (0)	
Tumor depth	*n*	30	16	14	
	mm, (mean ± SD)	8.7 ± 9.1	6.5 ± 8.4	11.2 ± 9.1	*ns (p = 0.06)*

Clinical parameters of skin squamous-cell carcinoma patients and specimens analyzed in this study. Categorial parameters in tumors with PNI and without PNI were compared using Chi-square test. NA: not available.

### Tumor infiltrating CD8+ T cell density is increased in the presence of high LEC density

**Figure 2:**
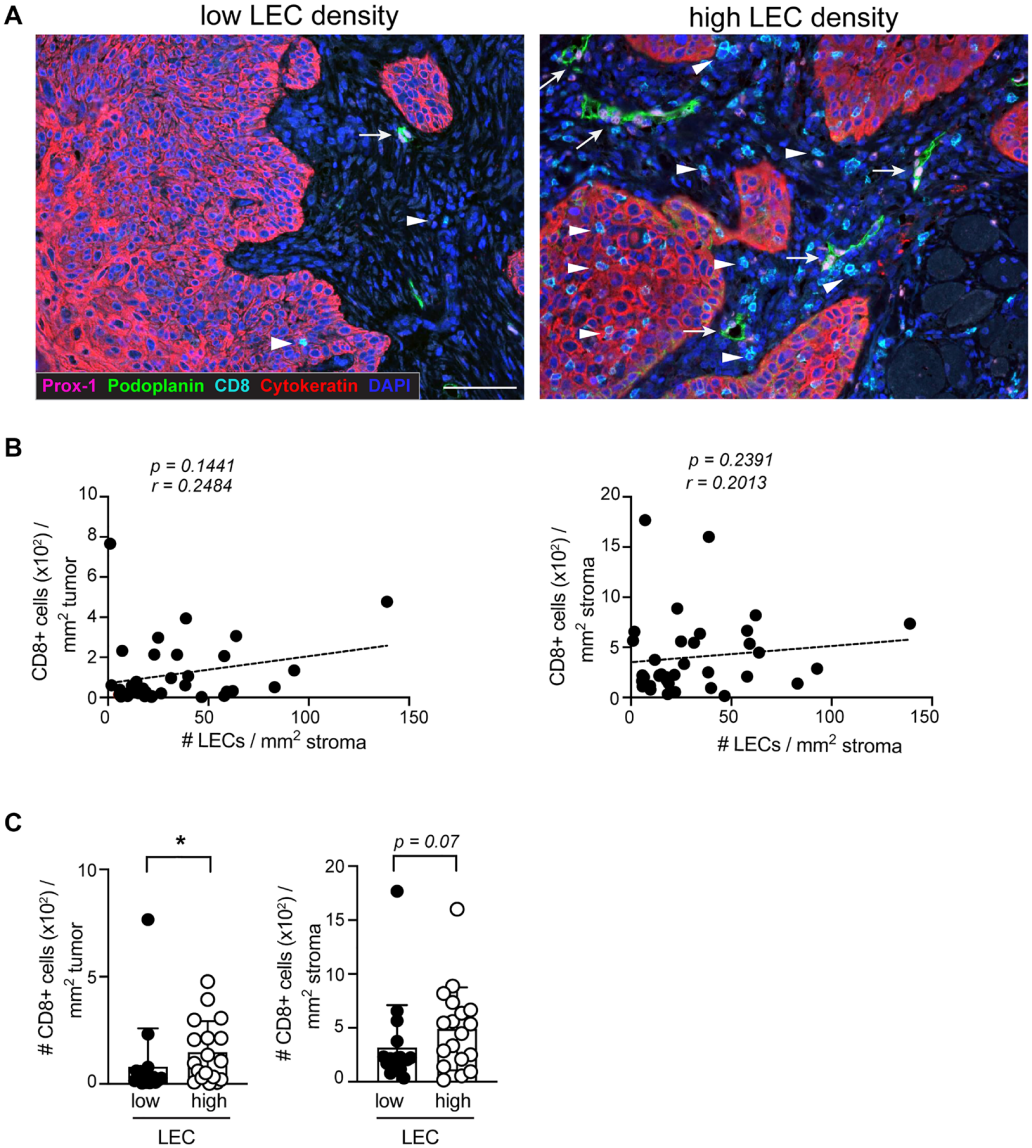
The density of CD8+ T cells in the tumor is increased in the presence of high LEC density. (**A**) Representative IHC images of sSCC sections illustrating lesions with high and a low LEC densities. Arrows indicate the location of LECs and arrowheads CD8+ T cells. Scale bar = 100 μm. (**B**) Scatterplot and dashed regression line showing the correlation of tumor and stroma infiltrating CD8+ T cells with LECs (*n* = 36). (**C**) Density of tumor (left) and stroma (right) infiltrating CD8+ T cells in sSCC categorized in high (*n* = 18) and low LEC (*n* = 18) groups based on below or above the median LEC density. Bar graphs showing mean ± SD. ^*^ *p* < 0.05.

Previous studies on melanoma have shown that the lymphatic vessel density (LVD) within the TME positively correlates with the abundance of stroma and tumor infiltrating CD8+ T cells, suggesting that the presence of lymphatic vessels favors an enhanced CD8+ T cell density within the tumor and the neighboring tissue [5, 21]. Indeed, even though high LVD is a poor prognostic factor in several cancers such as melanoma and breast cancer, it is predictive for favorable outcome to immunotherapy [22]. In our cohort, we did not find significant correlations of LEC density with stroma and tumor infiltrating CD8+ T cell density (Figure 2A and 2B). However, similar to the findings in melanoma, we found a significantly increased density of tumor infiltrating CD8+ T cells in samples with a high LEC density (Figure 2C).

Among the 36 sSCC samples analyzed, 11 tissue sections originated from solid-organ transplant recipients. Transplant patients are known to have a higher risk to develop certain types of cancer, linked to the treatment with immunosuppressive drugs. The incidence of sSCC as well as the development of aggressive sSCC has been shown to be elevated in transplant patients compared to the immunocompetent population [27, 28]. We determined frequency and distribution of CD8+ T cells and LECs and found slightly but not significantly reduced CD8+ T cell density in the tumor of transplant patients as compared to the non-transplant patients. Further, we found a similar LEC density in transplant patients compared to non-transplant patients (Supplementary Figure 2A and 2B).

### LEC and CD8+ T cell density is comparable in primary tumors from non-metastatic and metastatic sSCC

**Figure 3:**
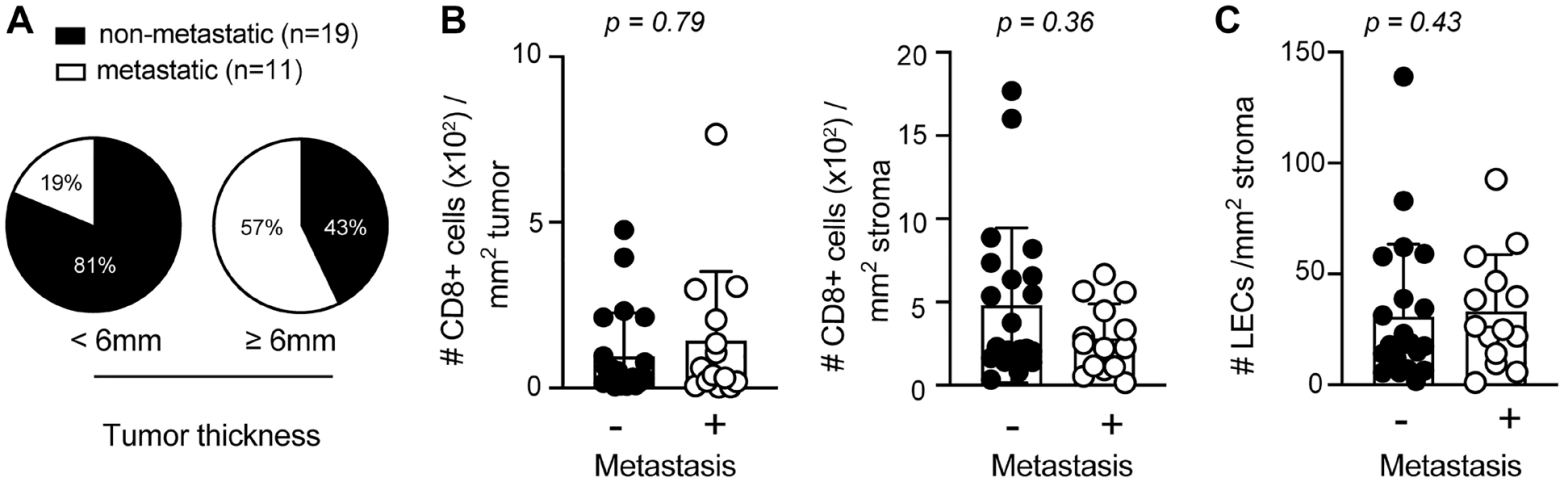
Elevated primary tumor thickness is more abundant in metastatic sSCC. (**A**) Pie charts indicating the percentages of sSCC patients with or without metastasis according to tumor thickness < (*n* = 16) or ≥ 6 mm (*n* = 14). Bar graphs indicating the frequencies of tumor (left) and stroma (right) infiltrating CD8+ T cells (**B**) and LECs (**C**) in sSCC with (*n* = 14) and without metastasis (*n* = 22). Bar graphs showing mean ± SD.

Tumor thickness and lymphatic vessel density have been identified as prognostic factors for tumor metastasis in sSCC [9, 23, 24]. In agreement with these findings, we found an increased likelihood for metastases in patients with a tumor thickness ≥ 6 mm (Figure 3A). In order to determine whether CD8+ T cell infiltration and/or LEC density are also predictive for metastatic disease, we compared the density of CD8+ T cells and LECs in sSCC sections from patients with (*n* = 14) and without metastases (*n* = 22). Interestingly, we did not find significant differences in stroma nor tumor infiltrating CD8+ T cells (Figure 3B). Previous studies found that tumor-associated lymphangiogenesis correlated with metastasis formation and poor clinical outcome in different types of human cancers [7]. However, we only found a trend but not a significant increase of LEC density with metastases (Figure 3C).

### Perineural infiltrated sSCC revealed a significantly lower LEC density

**Figure 4:**
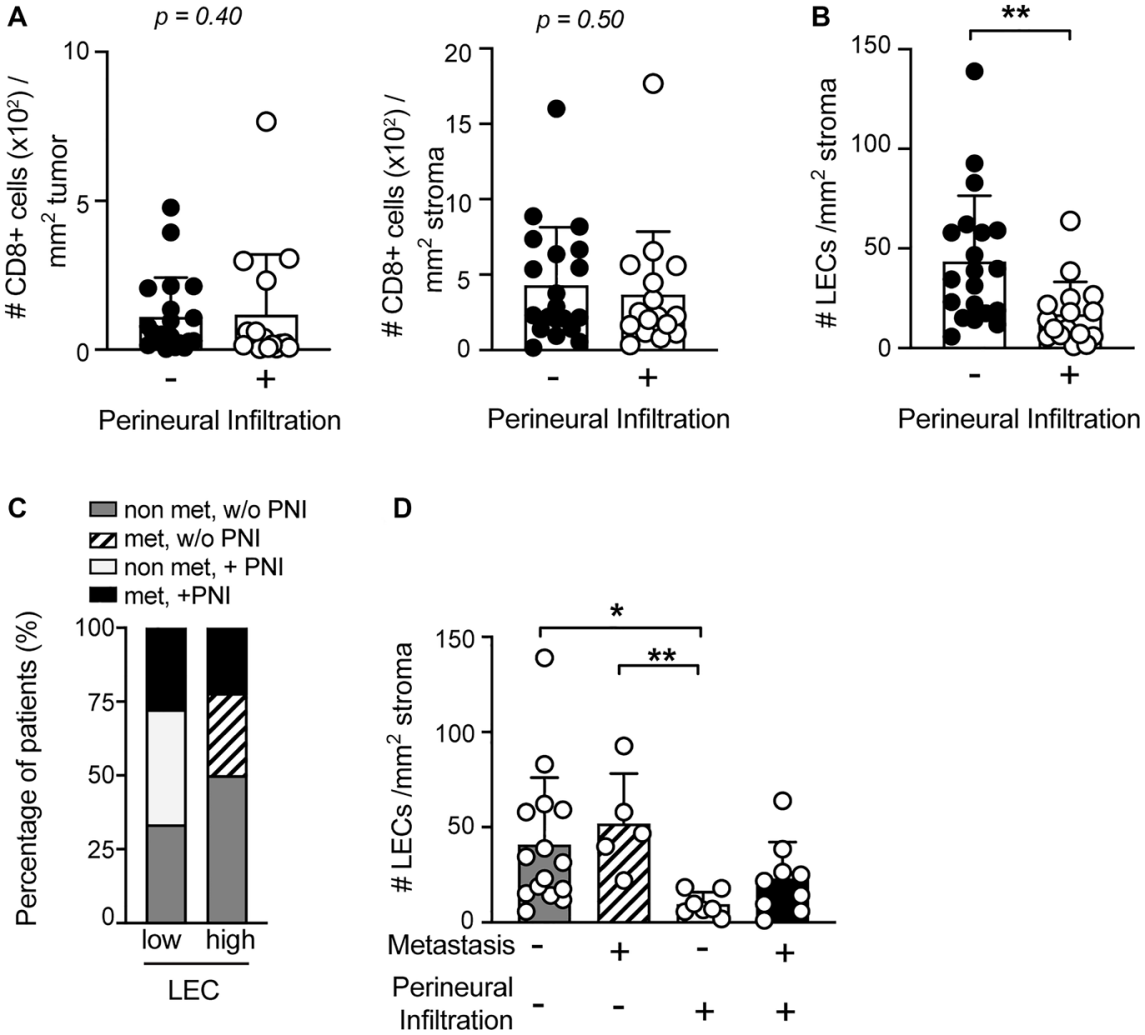
LEC density is significantly reduced in the TME of sSCC with perineural infiltration (PNI). The frequency of CD8+ T cells in tumor (left) and stroma (right) tissue (**A**) and of LECs (**B**) of primary sSCC sections were compared in patients with (*n* = 16) versus without PNI (*n* = 20). (**C**) Stacked bar graphs showing the distribution of the four sSCC patient groups plotted according to the presence of high or low LEC density in primary tumor tissue. (**D**) LEC density in primary sSCC without metastases (*n* = 15), sSCC with PNI (*n* = 7), sSCC with metastases (*n* = 5) and sSCC with both metastases and PNI (*n* = 9). Bar graphs showing mean ± SD. ^*^ *p* < 0.05, ^**^ *p* < 0.01.

Perineural infiltration (PNI) has been described as a characteristic of high aggressiveness in different cancer types and counts as a major risk factor, comparable to metastasis [27, 29]. sSCC sections were grouped according to the presence and the absence of perineural infiltration (PNI) (Table 1). Parameters including gender, iatrogenic immunosuppression, localization and patient age at diagnosis were comparably distributed in tumors with PNI and without PNI. The state of differentiation was significantly different in tumors with and without PNI, suggesting that sSCC cases with PNI tend to have a poor differentiation status. Furthermore, tumor depth showed a trend but was not significantly increased in sSCC with PNI (Table 1). To investigate whether the negative outcome of PNI might be linked to a low degree of CD8+ T cell infiltration in sSCC, we compared the CD8+ T cell density in the TME of perineural infiltrated to non-infiltrated tumor samples. Our data showed no significant difference in the density of neither stroma nor tumor infiltrating CD8+ T cells between the 2 groups of sSCC patients (Figure 4A).

Interestingly, we found a significant difference in the abundance of LECs in sSCC patients with PNI, compared to those without PNI (Figure 4B). Notably, perineural infiltrated sSCC tumors exhibited a significantly reduced LEC density. In order to evaluate this finding in conjunction with the metastasis status, we analyzed our patients in 4 different groups based on the absence or presence of metastases and/or PNI. Comparing the frequency of the different groups in samples with low and high LEC density, we found that all patients with PNI and no metastases were in the low LEC group (Figure 4C). Indeed, the TME of this patient group showed a very low density of LECs compared to the other groups (Figure 4D).

## DISCUSSION

Even though blood vessels are described as a main route of cancer cell dissemination [30], lymphatic vessels can exhibit a similar function by carrying cancer cells to the LNs and beyond within the lymphatic system [4, 31, 32]. Lymphatic vessels are known to be essential for tissue fluid homeostasis in the body and immune cell trafficking, but they are also actively involved in shaping the immune response. Lymphatic vessels located in the TME are activated which results in lymphatic vessel remodeling, similar as in inflamed tissues. Besides the structural remodeling, activated LECs also release factors such as IDO and NO that can dampen adaptive immune responses locally [17, 18]. This may play a role in the observed correlations of LVD in the TME with metastases and poor prognosis in several types of cancer [7, 23]. However, besides the pro-tumoral function recent studies revealed that high LVD is also associated with an enhanced immune cell infiltration and potentiates immunotherapy in melanoma [22]. In our study we investigated a putative link between the abundance of lymphatic vessels and tumor infiltrating CD8+ T cells in primary sSCC. Similar to previous data in melanoma [5], we found an increased frequency of intratumoral CD8+ T cells in sSCC with high LEC density, although there was in contrast to melanoma no significant positive correlation between LECs and CD8+ T cells. This suggests that the overall presence of CD8+ T cells in the TME of sSCC may be independent of LVD, but that high LEC density is predictive for an enhanced infiltration of CD8+ T cells into the tumor nests. In melanoma, high LVD is also associated with expression of immunosuppressive molecules and presence of regulatory T cells [5, 21], thus the presence of lymphatic vessels likely also contributes to immunosuppression. Upon immunotherapy, effector functions of CD8+ T cells are revitalized, thus possibly balancing those effects of lymphangiogenesis that counteract anti-tumor immunity. Consequently, it may be possible that an enhanced presence of lymphatic vessels is beneficial for sSCC patients treated with immunotherapy, similar to patients with melanoma.

Currently, several clinical trials are investigating anti-PD-1 therapy for patients with inoperable, recurrent or metastatic sSCC. The results of treatment with Cemiplimab show a response in almost half of the patients with metastatic sSCC [33]. A deeper understanding of factors contributing to immune responses in sSCC is needed in order to improve treatment indications based on the tumor biology. Our findings would suggest that the abundance of LECs in the peritumoral area could be a predictive factor for CD8+ T cell-based immunotherapy outcome.

The current literature on lymphatic vessels in sSCC focuses mainly on correlations with metastasis formation. Tumor associated macrophages present in the peritumoral area of sSCC express VEGF-C and are associated with increased LVD and lymphatic vessel reorganization [25]. Further, increased LVD was found in metastatic sSCC patients in comparison to non-metastatic patients when analyzing so-called lymphatic “hot spots” of peritumoral areas [24]. The cohort investigated in this study was selected based on two risk factors for sSCC, namely metastasis and perineural infiltration (PNI). Our data were generated on broad tumor and stroma areas selected independently of the presence of lymphatic “hot spots”. In contrast to the previous study by Krediet and colleagues [24] we did not find a significantly increased LEC density in the stroma of metastatic sSCC cases. Indeed, the different selection methods for the analyzed image fields as well as the relatively low number of sSCC lesions may have contributed to the discrepancy of results. Besides, previous findings in sSCC showed that tumor thickness is a predictive factor for metastases [24]. Similarly, we found that tumors with thickness of ≥ 6 mm were overrepresented in sSCC groups with metastases.

The degree of tumor differentiation was found to be associated with patient’s prognosis in sSCC [34, 35]. In our study we did not find any significant difference in CD8+ T cell infiltration and LEC density according to the state of tumor differentiation (data not shown), albeit the distribution of the different states varied significantly in cases with or without PNI. Similar to previous findings [36], most tumors from solid-organ transplant patients exhibited low infiltration of CD8+ T cells. However, our data did not show any significant difference in CD8+ T cell infiltration compared to tumors from non-transplant patients. Possibly, the low number of sections from transplant recipients in our analysis, as well as the frequency of so-called “immune desert” phenotype precluded the identification of a possible difference in intratumoral immune cell infiltration in patients with and without organ transplantation.

Neural cells in the TME have various functions and constitute an active area of cancer research. They promote the growth and the survival of cancer cells, for example in basal cell carcinoma [37, 38], and promote tumor invasiveness [39]. Interestingly, cancer cells have been shown to secrete neurotrophic factors (e.g., brain-derived neurotrophic factor) and to guide axons towards the tumor [40, 41]. Therefore, the cancer cell-nerve crosstalk promotes tumor progression and nerve sprouting [42]. In a similar way, cancer cells can produce VEGF-C and VEGF-D, promoting lymphangiogenesis and take advantage to disseminate via lymphatic vessels. Here, our data show a significantly lower LEC density in sSCC with PNI. Possibly, both PNI and lymphatic vessels have one or more common driver(s) that trigger(s) branching into perineural growth or lymphangiogenesis. This suggests that cancer cells can potentially favor PNI at the expense of lymphatic vessel sprouting in some cases. Considering the anti-apoptotic environment in the perineural space [38], neurotropism may be a more profitable alternative for cancer cell proliferation and spread than lymphotropism. The investigation of VEGF-C and neurotrophic factors may give insights into the underlying mechanisms governing the restricted amount of LECs in case of perineural infiltration.

Despite a lower LVD in perineural infiltrated sSCC, our data indicate no significant difference in CD8+ T cell infiltration according to PNI. As sSCC with PNI are known to be more aggressive and reduce patient survival [11, 13], we could speculate that CD8+ T cell function may be altered in tumors with PNI similar to findings in bile duct carcinoma [42]. Together our results suggest that lymphatic vessels play a role for the recruitment of immune cells to the tumor site in sSCC, as previously highlighted in melanoma [5, 22]. Further studies are required to confirm that the LEC density, in particular in patients with non-perineural infiltrated sSCC, may predict responses to current and novel therapies, including immunotherapy.

An entirely new observation is that perineural infiltrated sSCC in absence of metastases are characterized by low LVD compared to tumors without PNI. Since both are known to affect tumor progression and patients’ prognosis, it may be particularly important to identify the underlying mechanisms triggering either lymphangiogenesis or PNI, thus opening future options for therapeutic targeting.

## MATERIALS AND METHODS

### Patients

The skin squamous-cell carcinoma (sSCC) samples were collected and used in compliance with the study protocol n°2016-01851, approved by Commission cantonale d’éthique de la recherche sur l’être humain du canton de Vaud (CER-VD), in line with the legal requirements. 36 primary sSCC tumors from different patients were investigated 11 out of 36 patients were solid organ transplant recipients, of which 8 were involved in the Swiss Transplant Cohort Study [43].

Analyzed sSCC samples were classified in 4 groups: 15 sSCC without metastasis, 7 perineural sSCC without metastasis, 5 sSCC with loco-regional metastases (skin or LN) and 9 sSCC with loco-regional metastases (skin or LN) and perineural infiltration (in the primary tumor and/or metastases). The histopathological analysis of all tumors investigated in this study was verified by a board-certified dermatopathologist.

### Multiplex immunohistochemistry staining

3 μm thick tissue sections were cut from formalin-fixed and paraffin-embedded (FFPE) blocks of human primary sSCC excisions. Sections were deparaffinized followed by antibody labeling using the automated DISCOVERY ULTRA system (Ventana; Roche). The staining process is subdivided in sequential steps, consisting of a heat-induced antigen retrieval, followed by blocking, antibody labeling and detection by the Tyramide signal amplification (TSA) system. In between each labeling step antibodies were stripped of by a denaturation step. At the end sections were counterstained with DAPI (Biolegend) and mounted with fluorescence mounting medium (DAKO) [44]. Antibodies, TSA fluorescence systems and DAPI used are listed in the Table 2.

**Table 2:** Antibodies and fluorescent systems used in this study

Primary antibodies	Secondary antibodies	TSA fluorescence systems and counterstain
goat anti-human Prox-1 (polyclonal) - R&D Systems	rabbit anti-goat – HRP - Dako	TSA Cyanine 3 – Perkin Elmer
mouse anti-human Podoplanin (D2-40) - Biolegend	goat anti-mouse – HRP - Dako	TSA Cyanine 3.5 – Perkin Elmer
mouse anti-human Pan cytokeratin (AE1/AE3) - Dako	OmniMap anti-rabbit antibody – Ventana	TSA Cyanine 5.5 – Perkin Elmer
rabbit anti-human CD8 alpha (SP16) - Thermo Scientific	goat anti-rabbit – HRP - Dako	TSA FITC – Perkin Elmer
		DAPI - Biolegend

HRP: horseradish peroxidase, TSA: tyramide signal amplification.

### Multispectral imaging

All stained tumor sections were scanned at 10× magnification using Vectra 3.0 Automated Quantitative Pathology Imaging System (PerkinElmer). Using a whole slide viewer program (Phenochart 1.0.5; Perkin Elmer) multiple representative fields (up to 19.14 mm^2^) were selected within the tumor (defined by Pan cytokeratin+ cells and cell morphology) and the peritumoral stroma (1–3 mm thick region adjacent to tumor) [25]. The selected regions were imaged at a high magnification (20×) on a Vectra 3.0 Imaging System. Tumor and stroma areas were reviewed and approved by a board-certified dermatopathologist at the University Hospital of Lausanne.

### Computed quantitative image analysis

Image analysis of 20× images was performed with inForm 2.3 Advanced Image Analysis Software (PerkinElmer). All images were analyzed with the same successive steps: spectral unmixing and background subtraction, trainable tissue segmentation, cell segmentation, trainable phenotyping. To enable a color-based identification of the stains, the spectrum of each image was unmixed using a spectral library consisting of FITC, Cy3, Cy3.5, Cy5, Cy5.5 and DAPI single emission spectral curves. Background signal was defined on sSCC sections only probed with secondary antibodies and TSA and subtracted before further analysis. Automated tissue segmentation was performed based on DAPI and Pan cytokeratin-positive staining. Therefore, the program was trained to recognize Pan cytokeratin+ DAPI+ regions as tumor and Pan Cytokine - DAPI+ regions as stroma area respectively. Regions without any counterstain were defined as no tissue area and were not considered in further analysis steps. In all tissue-segmented images individual cells were segmented based on the DAPI counterstain using a cell segmentation algorithm. In order to distinguish different cell populations, we used the trainable phenotyping tool. To this end, lymphatic endothelial cells (Prox1+, Podoplanin+), CD8+ T cells (CD8+) and tumor cells (Pan cytokeratin+) were identified visually and assigned to the corresponding phenotype of interest. After this active learning step, a phenotyping algorithm that takes into account different parameters including fluorescence spectra and intensity, nuclear shape and cell morphology was generated and used to segment the different cell populations. The analysis method was reviewed and approved by two collaborators working at Centre des thérapies expérimentales (Department of Oncology, CHUV). All contributors to the imaging and analysis process were blinded for clinical and pathological information.

### Statistics

GraphPad Prism 8.4.3 was used to perform all statistical analyses. Nonparametric statistical tests and unpaired *t* tests were used for not normally distributed and normally distributed data sets, respectively. Mann-Whitney test was used to compare data from two groups and nonparametric Spearman correlation to investigate associations between two data sets. To compare multiple groups, we performed a Kruskal-Wallis test followed by Dunn’s multiple comparisons test. For clinical and histological parameters, a Chi-square test was performed to evaluate the equality of the distribution in samples with and without PNI. Two-tailed *P*-values < 0.05 were considered significant.

## SUPPLEMENTARY MATERIALS



## Abbreviations

FFPE: Formalin-fixed and paraffin-embedded
IDO: Indoleamine-2,3-dioxygenase
LEC: Lymphatic endothelial cell
LN: lymph node
LVD: Lymphatic vessel density
NO: Nitric oxide
PNI: Perineural infiltration
PD-1: Programmed death-1
PD-L1: Programmed death-ligand 1
sSCC: Skin squamous-cell carcinoma
TME: Tumor microenvironment
TSA: Tyramide signal amplification
VEGF: Vascular endothelial growth factor
