# Addendum: Mutually exclusive lymphangiogenesis or perineural infiltration in human skin squamous-cell carcinoma

**DOI:** 10.18632/oncotarget.28319

**Published:** 2022-12-06

**Authors:** Julien Schaller, Hélène Maby-El Hajjami, Sylvie Rusakiewicz, Kalliopi Ioannidou, Nathalie Piazzon, Alexandra Miles, Déla Golshayan, Olivier Gaide, Daniel Hohl, Daniel E. Speiser, Karin Schaeuble

**Affiliations:** ^1^Department of Oncology, University Hospital Lausanne (CHUV/UNIL), Lausanne, Switzerland; ^2^Institute of Pathology, University Hospital Lausanne (CHUV), Lausanne, Switzerland; ^3^Department of Dermatology, University Hospital Lausanne (CHUV), Lausanne, Switzerland; ^4^Transplantation Center, University Hospital Lausanne (CHUV/UNIL), Lausanne, Switzerland

**This article has an addendum:** The skin squamous-cell carcinoma (sSCC) samples were collected and used in compliance with the study protocol n°2016-01851, approved by Commission cantonale d’éthique de la recherche sur l’être humain du canton de Vaud (CER-VD), in line with the legal requirements. Patients signed a general consent for use of health-related data.

Original article: Oncotarget. 2021; 12:638–648. 638-648. https://doi.org/10.18632/oncotarget.27915

