# Supplementary material for: Mutually exclusive lymphangiogenesis or perineural infiltration in human skin squamous-cell carcinoma
Source: Oncotarget. 2021 Mar 30;12(7):638–48. doi: 10.18632/oncotarget.27915 (PMC8021034; doi:10.18632/oncotarget.27915)
Supplement: Supplementary file 1 [file oncotarget-12-638-s001.pdf]

Mutually exclusive lymphangiogenesis or perineural infiltration in human skin squamous-cell carcinoma

SUPPLEMENTARY MATERIALS

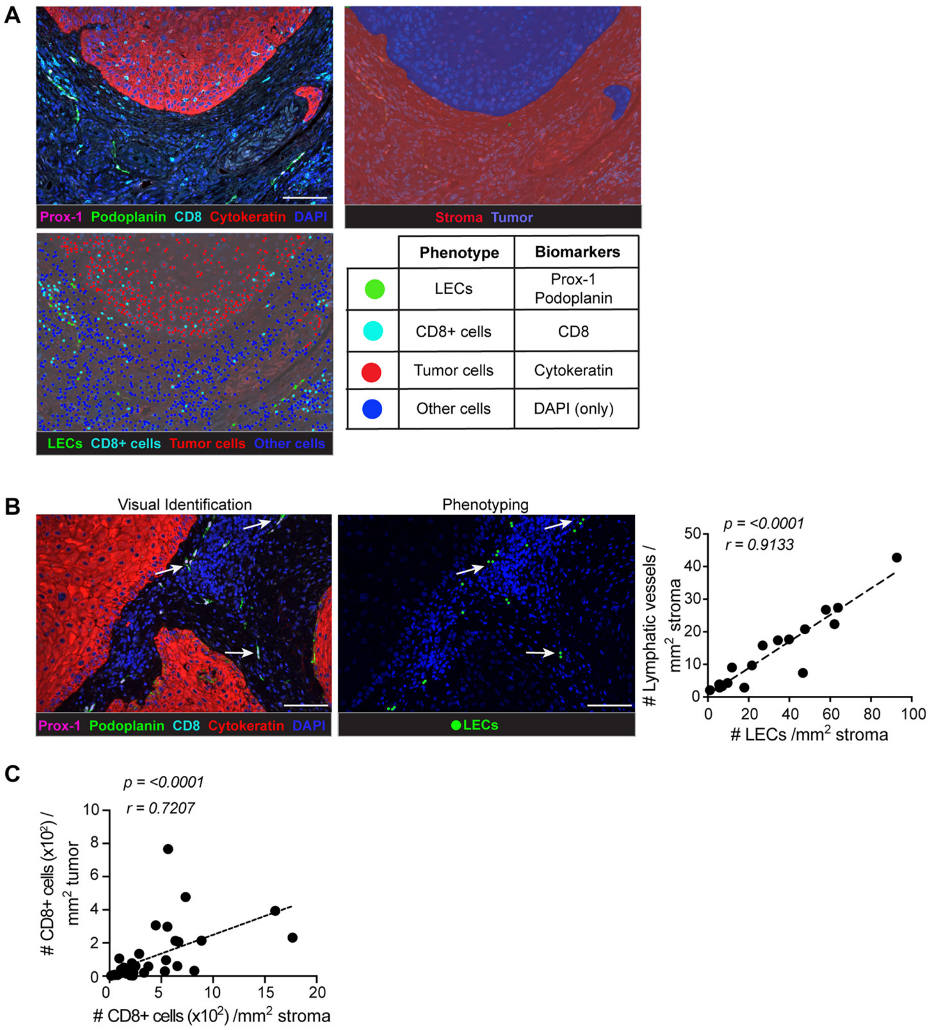

Supplementary Figure 1: Quantification of LECs by trainable image analysis correlates with visual lymphatic vessel identification. (**A**) Representative IHC image of sSCC section stained for LECs (Prox-1+ Podoplanin+), CD8+ T cells (CD8+) and tumors cells (Cytokeratin+). Tissue segmentation into stroma (red) and tumor (blue) areas was done based on Cytokeratin and DAPI labeling and followed by cellular phenotyping in order to discriminate indicated cell populations. (**B**) Representative IHC images showing lymphatic vessels identified visually and by trainable phenotyping. Scatterplot with dashed regression line depicting the correlation of LEC density determined by quantitative image analysis and the lymphatic vessel number counted by visual investigation. (n = 18) (**C**) Correlation of tumor and stroma infiltrating CD8+ T cell density determined in 36 sSCC samples. Scale bars = 100 µm.

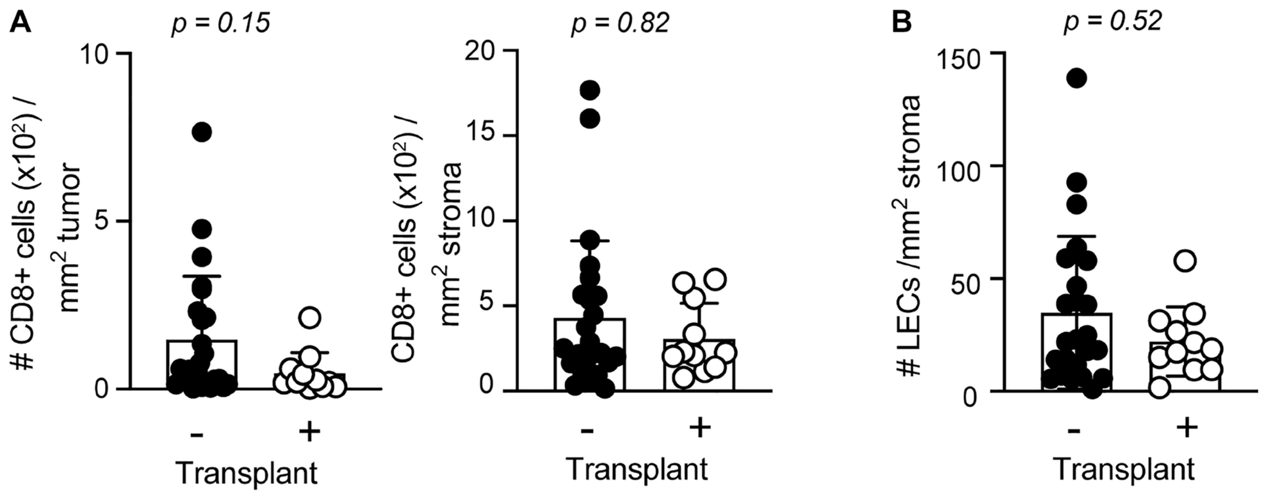

Supplementary Figure 2: Comparison of LEC and CD8+ T cell densities in sSCC sections from non-transplant versus transplant patients. Bar graphs showing the density of tumor and stroma infiltrating CD8+ T cells (**A**) and LECs (**B**) in primary sSCC sections from transplant (*n* = 11) and non-transplant (*n* = 25) patients. Bar graphs showing mean ± SD.
